# How Effective Are Spiritual Care and Body Manipulation Therapies in Pediatric Oncology? A Systematic Review of the Literature

**DOI:** 10.5539/gjhs.v6n2p112

**Published:** 2013-12-10

**Authors:** Thomas G. Poder, Renald Lemieux

**Affiliations:** 1 UÉTMIS – CHUS, Sherbrooke, QC, Canada; 2 CRC Etienne-Le Bel, Sherbrooke, QC, Canada; 3 DSQ – DGTI – MSSS, Montréal, QC, Canada

**Keywords:** complementary and alternative medicine, spiritual care, body manipulation, pediatric, oncology

## Abstract

**Background:**

The effects of cancer and associated treatments have a considerable impact on the well-being and quality of life of pediatric oncology patients. To support children and their families, complementary and alternative medicines are seen by nurses and doctors as practical to integrate to the services offered by hospitals.

**Objective:**

The purpose of this paper is to examine if the practice of complementary and alternative medicine, specifically spiritual care and treatments based on body manipulation, is likely to improve the health and well-being of children suffering from cancer.

**Method:**

This objective is achieved through a systematic review of the literature. The level of evidence associated with each practice of complementary and alternative medicine was assessed according to the methodological design used by the studies reviewed.

**Results and Conclusion:**

Studies reviewed are of a methodological quality that could be described as fair due to the small sample size of patients and the existence of a number of biases in the conduct and analysis of these studies. However, results obtained are consistent from one study to another, allowing us to make certain recommendations. It is thus advisable to consider the introduction of hypnotherapy in pediatric oncology services. Based on the data collected, it is the complementary and alternative medicine with the most evidence in favor of effectiveness of the well-being of pediatric oncology patients, especially during painful procedures. It is also recommended to use art therapy and music therapy. Conversely, too little evidence is present to be able to recommend the use of acupuncture, chiropractic or osteopathy.

## 1. Introduction

Today, the five year survival rate for children with cancer is above 80% (Jemal et al., 2008). This success is due largely to invasive treatments with and without significant side effects. This results in pain, anxiety and distress (Kuppenheimer and Brown, 2002; Salas Arrambide et al., 2002; Zernikow et al., 2005). In order to relieve these patients, some studies have suggested the advantages of using complementary and alternative medicine (CAM) (Sencer and Kelly, 2007; Landier and Tse, 2010). These approaches are primarily holistic in nature and focus on wellness and healing powers rather than the disease. Strengthening the use of CAM could thus be a beneficial avenue for all to the extent that the integration of CAM in the treatment plan of the child may: 1) strengthen the sense of control in the patient and his family; and 2) promote a sense of active participation and partnership with the nursing staff throughout the healing process (Sencer and Kelly, 2007). In addition, CAM can initiate ideas and innovative approaches that are a potential source of effective therapeutic treatments while reducing certain undesirable side effects.

### 1.1 CAM and Its Use in Pediatric Oncology

The definition of complementary and alternative medicine adopted here is that of the National Center for Complementary and Alternative Medicine (NCCAM): “a group of diverse medical and health care systems, practices, and products that are not generally considered part of conventional medicine” (NCCAM, 2012). This definition thus refers to the use of non-mainstream approach together with or in place of conventional medicine. Moreover, in many cases, CAM helps manage symptoms and side effects of medical treatments. In such cases, various practices with origins outside of mainstream medicine (CAM) are integrated into treatment and health promotion (i.e. integrative medicine).

According to the NCCAM (2012), there are three broad categories of CAM: natural products, spiritual care (mind-body) and treatments based on body manipulation.

The first category of CAM uses a variety of herbal medicines, vitamins, minerals, and other “natural products.” Many are known as dietary supplements and it also includes probiotics.

Spiritual care (mind-body) focuses on the interactions between the brain, mind, body and behavior, aiming to use the mind to affect physical functioning and promote health. Many CAMs include this concept: meditation, yoga, acupuncture, hypnotherapy, TAI CHI, imagery, etc. However, spiritual care is much more than this utilitarian definition. Indeed, spirituality is often described as a dimension of a person that involves meaning, purpose, transcendence, connectedness and energy (Chiu et al. 2004, Pesut et al. 2008). In this setting, religion is a subset of spirituality (Hollins 2005) and has been defined as an institutional body of beliefs and practices (Chiu et al. 2004). Despite these distinctions, these different concepts are often not distinguished in the literature, which may explain why there has been little consensus on the concept of spiritual care.

Care based on body manipulation mainly focuses on the structures and systems of the body, including bones and joints, soft tissues and the circulatory and lymphatic systems. The three most important therapies in this group are spinal manipulation (chiropractic), manipulation of the musculoskeletal and myofascial system (osteopathy) and massage therapy.

These practices are widespread in North America and Europe and some studies estimate that 80% or more of patients (adult and pediatric) with cancer use CAM (Roberts et al., 2006; Paisley et al., 2011). Specifically, the investigation by Paisley et al. (2011) indicates that 82% of pediatric oncology patients whose treatment in chemotherapy has failed at least once use CAM. This is mainly spiritual care (83%) and dietary supplements (31%). The same study also shows that 60% of CAM users did not inform their oncologist of such a practice (maybe because they do not ask it), which is potentially dangerous in the event of interactions or side effects from the combination of CAM with chemotherapy (Roth et al., 2009; Chiu et al., 2009). This very high rate of use of CAM is mainly due to the finding of a failure of chemotherapy and disease recurrence (Martel et al., 2005). Similarly, the study by Fouladbakhsh et al. (2005) demonstrates that patients with advanced stage cancer are more likely to use CAM. According to studies, there is considerable variability of rates of use of CAM in pediatric oncology, ranging from 6% to 91% (Sencer and Kelly, 2007; Bishop et al., 2010). However, over half of these studies report rates between 20% and 60% (Bishop et al., 2010). By comparison, the study by Post-White et al. (2009a) reported that, in a sample of 281 patients, 59% of pediatric patients with cancer use CAM, compared to only 36% of pediatric patients without cancer.

In the systematic review of the literature by Bishop et al. (2010), which does not distinguish between pediatric patients who have had a failure in their cancer treatment or not, it is stated that the CAM most commonly used are herbal therapies, dietary supplements and spiritual care. In this review, the use of medicinal plants (herbal therapy) ranged from 2% to 48%, the use of dietary and nutritional interventions ranged from 3% to 47% and the use of faith healing therapies ranged from 3% to 30% for children surveyed. Besides these three main categories of CAM, four are reported in fewer studies: homeopathy was used by 1% to 17% of patients in seven studies; megavitamins were used by 2% to 19% of patients in seven studies; the mind-body therapies (excluding prayer spiritual care) were used by 9% to 27% of patients in five studies; finally, massage therapy has been used by 2% to 17% of patients in five studies. The review by Bishop et al. (2010) also reported a trend likely to increase the use of CAM in time, especially for herbal therapies and faith healing. A significant positive association between the level of parental education and the use of CAM is also reported (5 out of 14 studies).

### 1.2 Research Question

This study seeks answers to the following question through a systematic review of scientific literature:

“Is the practice of complementary and alternative medicine likely to improve the health and well-being of children suffering from cancer? If so, which ones appear to be most relevant to use on the basis of available scientific data?”

## 2. Research Methodology

In this paper, we performed a systematic review of the literature. To do so, we considered the methodological design of each study to establish the level of evidence of the same.

Search engines used for this systematic review are: Embase, CINALH, AMED, OVID Healthstar, OVID Medline, Mantis, Pubmed and ScienceDirect. The websites of the National Institute of Excellence in Health and Social Services, the NIHR Health Technology Assessment Programme, the CMA Infobase and the Directory of Good Practice Recommendations and French-speaking consensus conferences were also consulted.

The research of articles was conducted over a period from January 1975 until January 2012. The languages used for this research are English and French.

Key words used in different search engines are:

- oncology / oncologie;
- pediatric / pédiatrie;
- child / enfant;
- cancer;
- alternative medicine / médecine alternative;
- complementary medicine / médecine complémentaire;
- art therapy / art thérapie;
- music therapy / musicothérapie;
- mind-body therapy / thérapie corps-esprit;
- massage or reflexology / massage ou réflexologie;
- hypnosis / hypnose;
- acupuncture / acupuncture;
- aromatherapy / aromathérapie;
- chiropractic / chiropractie;
- osteopathy / ostéopathie.

These keywords where combined in English or in French in sets of 3-4 words in order to trace article about CAM in pediatric oncology. For example, we used “oncology” AND “pediatric” AND “music therapy”.

Any study of the effectiveness, quality of life and well-being of patients in pediatric oncology, through the use of CAM, was chosen. Studies involving non-human subjects were excluded. To the extent that in general and especially in our hospital, doctors and nursing staff strongly recommend that patients and their families avoid supplements, herbs, natural medicine, homeopathy and others that may interact with chemotherapy agents which are recognized and proven effective against the disease, these groups of CAM were excluded from our research (Weitzman, 1998; Cassileth & Deng, 2004).

The two areas of interest selected for this review of literature are spiritual care (meditation, imagery, prayer, art and music therapy, acupuncture and hypnosis) and treatments based on body manipulation (massage with or without essential oils, chiropractic and osteopathy).

In fact, our criteria for inclusion are: Studies in French and English; Studies on human populations; Literature reviews; Primary studies; Studies using CAM; Studies investigating the effect of CAM on the well-being, anxiety, pain and side effects of cancer treatments. Our exclusion criteria are: Studies with adults; Studies with dietary supplements (supplements, herbs, natural medicine and homeopathy).

Papers included were preselected on the basis of their content summaries and title. If the content was about pediatric oncology and CAM, the article was selected for a complete reading. After reading the entire article, articles were included if they corresponded to our inclusion and exclusion criteria. Divergence between authors during this process did not occur; otherwise someone else would have proceeded to arbitration.

The level of scientific evidence of the studies included for each CAM refers to the number of randomized studies according to the number of patients recruited. Thus, the level of overall evidence for studies converging towards the same results has been established by the work group as follows: 1) good if 5 or more RCT with n ≥50; 2) good to fair if 5 or more RCT with n<50; 3) fair if 1-4 RCT, regardless of the value of n; 4) weak, if otherwise. This choice was based on the fact that it is difficult to use a unique appraisal tool for different design of study and because different tools give the higher level of evidence to large randomized studies (Hailey et al., 2002; HAS, 2010).

## 3. Results

The research carried out was able to identify 681 studies, of these seven reviews of literature, four meta-analyses and 42 primary studies were selected. The studies excluded were done so based on the exclusion criteria established in Section 4.

### 3.1 General Results from the Systematic Review by Landier and Tse (2010)

The most recent and most comprehensive of reviews on the effects of CAM on pain, anxiety and distress in children treated for cancer is the one carried out by Landier and Tse (2010). This review of literature excludes the application of CAM on surgical procedures and only involves the following medical procedures: bone marrow aspiration, lumbar puncture, injection and venipuncture. The population selected is from the age of 2 to 18 years. The results associated to the parents were not listed. All studies analyzed (n=32) refer to CAM based on the mind-body approach and in particular distraction techniques (18/32), hypnosis (11/32) and imagery (6/32).

In this review, it is reported that distraction is particularly effective for young children. Distraction techniques refer to counting, singing, watching a video, playing a virtual reality game or simply drawing attention away from the child’s medical procedure. As regards to hypnosis, this is a more structured procedure in which a patient is guided by a therapist to respond to suggestions for changes related to a subjective experience (such as alterations of perception, emotion, thinking, behavior and sensation). According to Accardi and Milling (2009), responsiveness to hypnosis increases from the age of 3, and a peak is reached between the ages of 8 and 12 years, then this ability decreases to the age of 16 years, after which it tends to remain stable for life. Imagery, in turn, focuses the attention of the child away from the medical procedure by taking advantage of the imagination. For example, the child may be asked to imagine himself in a nice place and focus on the physical sensations he can experience in the imaginary place. This technique requires the active cooperation of the patient and is most effective when used with children over 8 years of age (Doellman, 2003). In the studies listed by Landier and Tse (2010), the imagery technique is often used in combination with distraction, relaxation and games.

To relieve symptoms of pain, anxiety and distress during medical procedures for the treatment of children with cancer, it appears that hypnosis may be more effective than distraction, in particular for highly painful procedures such as lumbar punctures and bone marrow aspirations (Zeltzer and LeBaron, 1982). Among the 32 studies analyzed by Landier and Tse (2010), except for the study by Manne et al. (1990) who reported no effect, all indicate a positive impact of the CAM, sometimes with a significant statistic. The majority of these studies used small sample sizes and certain interventions include several CAM, which does not distinguish the effectiveness of each (in particular distraction and imagery).

In summary, all studies indicate a positive impact of CAM, but often without statistical reference. This observation, together with the small sample sizes, reduces the level of evidence to “fair” in most studies. It appears that hypnosis is more effective than other CAM to relieve symptoms of pain, anxiety and distress during medical procedures. This technique would be more effective in children aged 8 and over. In turn, the “distraction” method would be particularly effective in young children.

### 3.2 Hypnosis

As mentioned in the systematic review of the literature conducted by Landier and Tse 2010, changes in perceptions and sensations occur during hypnosis. Although the exact mechanism of action is not yet well established, work in neuro-imaging show that hypnosis is associated with an activation of brain areas corresponding to a decrease in arousal, an increase of visual imagery and a possible reinterpretation of the perceptual experience (Rainville et al., 2002; Wood and Bioy, 2008; VanHaudenhuyse et al., 2008).

Hypnosis can be informal, for example, by inviting a subject to listen to or participate in a story or a fairy tale, or formal, by inviting the subject to stare at the hypnotherapist while he makes suggestions. In general, hypnosis can be used for several purposes: 1) to reduce anticipated stress; 2) to develop coping strategies; 3) to block or manipulate the sensation of pain.

The more the child is susceptible to hypnosis, the greater the effect may be. The sensitivity of the child to hypnosis can be measured by several scales, with the most used in clinical settings being the “Shorter Stanford Clinical Scale for Children” (Morgan and Hilgard, 1978-1979).

Apart from studies listed by Landier and Tse (2010), the study by Smith et al. (1996) shows that hypnosis is more effective than distraction in children with cancer highly susceptible to hypnosis in order to reduce pain and anxiety during treatments such as venipuncture, lumbar punctures or bone marrow aspiration. In the case of children less susceptible to hypnosis, distraction appears to be equally effective. In the study by Liossi and Hatira (2003), both forms of hypnosis (formal or informal) lead to comparable results and the level of sensitivity to hypnosis was significantly associated with the benefits of hypnosis. In contrast, self-hypnosis provides fewer benefits.

In general, the review of literature by Richardson et al. (2006) specific to hypnosis concluded that it provides benefits to the pediatric cancer patients as regards to the reduction of pain, anxiety and distress during painful procedures, and that these benefits are quite similar to those obtained from the use of games and distraction. This result does not however take into account two recent studies by Liossi et al. (2006, 2009) that indicate a superiority of hypnosis over simple distraction. Moreover, as in the study by Liossi and Hatira (2003), Richardson et al. (2006) indicate that hypnosis is potentially more efficient with the help of a therapist rather than carried out alone.

A second review of literature by Richardson et al. (2007) studies the impact of hypnosis on nausea and vomiting induced by chemotherapy. This review indicates significantly positive effects of hypnosis. The effects of hypnosis are evaluated as more important compared with standard therapy (i.e. distraction, conversation, breathing control), moderate compared to touch therapy and slight compared to cognitive behavioral therapy.

In summary, hypnosis is commonly used to reduce pain, anxiety and distress during painful procedures, but also to reduce chemotherapy-induced vomiting. The more the child is susceptible to hypnosis, the more the effect of the latter is important. The child’s sensitivity to hypnosis can be measured with several scales; the most used is the “Shorter Stanford Clinical Scale for Children.” The number and quality of the studies reviewed indicate a level of evidence of “good to fair”.

### 3.3 Massage Therapy

Although massage therapy provides a promising option for children with cancer, few data are available on this population. However, research in other pediatric populations suggest that massage is an effective treatment, including for premature infants and infants exposed to HIV, as well as for children with asthma, cystic fibrosis, diabetes and rheumatoid arthritis (Beider and Moyer, 2007; Cassileth and Vickers, 2004; Field et al., 1997a, 1997b, 1998; Hernandez-Reif et al., 1999; Scafidi et al., 1990). Documented results include weight gain, reduced cortisol levels, decreased anxiety and depression, improved sleep, improved immune function and reduced pain. In the case of cancer patients, the study by Quattrin et al. (2006) indicates that foot massage during chemotherapy led to a decrease in anxiety in adult patients. More generally, in the systematic review of Wilkinson et al. (2008) including only randomized studies, it is suggested that massage in adults with chemotherapy may reduce anxiety in the short term and possibly reduce symptoms of pain and nausea. Similar results have been reported by the non systematic review by Myers et al. (2008) on massage, reflexology and acupressure as well as the review of literature by Hughes et al. (2008). Moreover, the latter provides an update on various existing massage techniques. The main type of massage practiced is Swedish massage. Other types of massage are reflexology, shiatsu, acupressure, manual lymphatic drainage and Ayurvedic massage.

For its part, the review by Field et al. (2005) indicates that, in adults and children, cortisol decreases and serotonin and dopamine increase after a massage. This result for cortisol is not supported by the meta-analysis by Moyer et al. (2011) in adults (non-significant results with little variation) and only very few studies with children seem to show such an effect. Considering the number of studies indicating a decrease in anxiety following massage, it therefore appears that the decline in cortisol is probably not the cause of this result. In addition, these reviews do not mention the potential bias induced by treatment with corticosteroid in the evolution of cortisol.

With regard specifically to children with cancer, we identified three studies regarding massage therapies. A randomized study in 23 children from 1-18 years of age, recruited from two oncology clinics in Minnesota (Post-White et al., 2009b), compared massage sessions with periods of silence/rest over a period of 4 weeks of treatment. This study indicates that the practice of massage reduces anxiety in children aged 14 and under and their parents. In these children, massage therapy also reduces the heart rate. In contrast, no changes are reported in terms of blood pressure, cortisol, pain, nausea and fatigue. The children also indicate that the beneficial effect of massage lasts longer than that associated with periods of silence/rest. On the other hand, another randomized study, by Phipps et al. (2010), with 178 children found no significant difference over a period of six weeks between groups with or without massage during treatment with stem cell transfer. Finally, another randomized study (Haun et al., 2009) on 30 children between the ages of 6 months and 17 years with cancer or blood diseases showed that the group which received four massage sessions (daily for hospitalized patients, weekly for the others) showed significantly better results than the group without massage regarding anxiety, emotional state, muscle pain, discomfort and respiratory rate.

In summary, the technique of massage is recognized in studies for the reduction of anxiety levels, but to varying degrees. While some studies show a physiological change associated with massage (i.e. decrease in cortisol, increased levels of serotonin and dopamine), other studies did not show and relate rather a feeling of anxiety reduction. The level of evidence from these studies is considered here as “fair”.

### 3.4 Aromatherapy

Aromatherapy is the use of essential oils from plants for healing. Only one study addressing the effects of aromatherapy was listed in pediatric oncology (Ndao et al., 2010). This study involves children and adolescents undergoing stem cell infusion. The study was conducted double-blind with a placebo (shampoo fragrance). The aromatic agent was bergamot (citrus X bergamia) and its effect on anxiety, nausea and pain in 37 patients was studied. The patients were evaluated upon recruitment, before stem cell infusion (but after a drug infusion), upon completion of the infusion and one hour later. The results indicate that patients in the treatment group expressed more anxiety (p = 0.05) and reported more nausea (p = 0.03) an hour after the infusion than the control group. In both groups, nausea and pain decrease, however, following the intervention. In contrast, anxiety in children and adolescents only decreased significantly in the control group. This study demonstrates that the use of essential oil with bergamot does not have a more significant anti-anxiety effect than just fragrance. In fact, a pleasant smell, whatever its nature, has a positive effect in children. Similar results were found in a study on adults (Graham et al., 2003). Ndao et al. (2010) also report that the kind of essential oils chosen for their study may not be appropriate for a mainly male public (73% of cases in this study) and who might prefer a non citric essence, as reported by Fitzgerald et al. (2007). In addition, the inhaled dose also influenced the results. Indeed, if the dose is too high it will have a more stimulating than sedating effect (Bagetta et al., 2010). Bagetta et al. (2010) also suggest that the effect of the combination of essential oils with a massage would be more efficient by allowing absorption in the peripheral tissues, causing a reduction in pain sensitivity. For example, the study by Wilkinson (1995) randomly divided 51 patients among a group with massage and without aromatherapy and a group with massage and aromatherapy. Three massages were given during one week. The results indicated an improvement in both groups compared to the baseline, in contrast only improvement in the group with aromatherapy is statistically significant with regard to quality of life (p=0.005) and physical impact (p=0.003). On the other hand, patients perceived massage with aromatherapy as being able to reduce tension, pain and anxiety. Similar results were found in the study on a larger scale by Wilkinson et al. (2007) (n=288). A systematic review by Fellowes et al. (2004) also considers some studies combining massage and aromatherapy and suggests superior efficacy of massage with aromatherapy versus massage alone, particularly with regard to anxiety and quality of life. All studies, except the one by Ndao et al. (2010), only involve adults however, which does not allow us to generalize these results given the differences that may exist with the pediatric patients.

In summary, only one study on the use of aromatherapy in pediatric oncology has been reported. This study was randomized and the level of evidence for aromatherapy is considered “fair”. Here it is shown that the use of essential oil with bergamot has no more anti-anxiety effects than a simple fragrance. On the other hand, in adults, where studies are more numerous, the combination of aromatherapy and massage increase the effectiveness of the massage.

### 3.5 Osteopathy and Chiropractic

No studies linking treatment in pediatric oncology with osteopathy or chiropractic has been reported. However, there are studies for adults as well as for children having survived cancer (Alcantara et al., 2011; Montgomery et al., 2011). These studies indicate that physical therapy and chiropractic are widely used in patients with cancer. According to Evans and Rosner (2005), the judicious use of chiropractic services may correspond to economic and effective strategies to reduce pain and suffering, with the potential to improve the overall health of the patient. However, this is primarily a qualitative rather than quantitative analysis (no specific quantitative data to support this). Furthermore, it is recommended that a comprehensive assessment of the patient with cancer be carried out in order to determine the potential contraindications to the use of chiropractic. Alcantara et al. (2011) have listed four case studies in which chiropractic was potentially the cause of an adverse event. Consequently, it is useful to describe, as does Shaw (2007), what the chiropractor needs to know and do as well as what should be avoided for the well-being of cancer patients.

In summary, no study of the use of chiropractic or osteopathy in pediatric oncology has been reported. In other patient populations, these techniques could be effective in reducing pain and suffering if used judiciously. Otherwise, side effects could potentially occur.

### 3.6 Acupuncture

The mechanisms of action of acupuncture are subject to considerable debate. The main explanation found in literature indicates that acupuncture releases neurochemicals in the body, such as endorphins, enkephalins and serotonin (Moffet, 2006). Another theory suggests an effect on the sympathetic and parasympathetic nervous system (Haker et al., 2000).

In general, there are few studies investigating the effect of acupuncture on children in oncology. Maybe this is because children are often afraid of needles and the pediatric oncology population is very vulnerable. Regarding the pediatric population in general, Kemper et al. (2000) indicate, however, that 67% of children referred for acupuncture for chronic pain have found the experience positive. Moreover, the question can also be asked whether the technique is appropriate for individuals who have not completed their physical development, especially regarding the placement of needles at or near the fontanel.

The review of literature conducted by Jindal et al. (2008) refers to all children, not only to the pediatric oncology population. This review of literature lists 23 randomized studies, six systematic reviews and two meta-analyses. In most cases, the control group consists of a simulation of acupuncture. In addition, they indicate that “non-invasive modalities, such as electrical stimulation or laser, on acupoints and acupressure seem to be well accepted by younger children” (Jindal et al., 2008, p.3). The results indicate evidence in favor of a certain efficacy and low risk associated with acupuncture in pediatrics. As regards to pediatric oncology patients, evidence of effectiveness is less clear than in other pediatric specialties, such as postoperative management. In this review of literature conducted by Jindal et al. (2008), the rate of adverse events associated with acupuncture is estimated at 1.55 for 100 acupuncture treatments (i.e. redness and pain are the most common). The rate of serious adverse events is, meanwhile, estimated at 5.36 per 10,000 treatments. These rates are not based on formal cause and effect relations, but rather on certain assumptions made in the studies reviewed by Jindal et al. (2008). These rates could be even lower. In addition, adverse events were not related to a specific category of acupuncture (i.e. invasive or non-invasive).

Studies specifically related to acupuncture and pediatric oncology are three in number. In the first, by Reindl et al. (2006), there is no statistically significant difference between the group without and the group with acupuncture with regard to vomiting. In contrast, in the group with acupuncture, there was a significant decrease in consumption of antiemetics given in excess of the base level (i.e. rescue antiemetics). The same result is obtained in the study by Gottschling et al. (2008). In this study, the authors also found a decrease in vomiting episodes with acupuncture versus no acupuncture. In an unpublished pilot study, but reported by Reindl et al. (2006), it is also mentioned that acupuncture has a relaxing effect by causing drowsiness and a reduction in heart rate.

In summary, the level of effectiveness of acupuncture in the pediatric community is variable, but positive, in particular regarding the reduction of pain and vomiting. The adverse event rate remains below 1.6% and more specifically, the rate of serious adverse events is less than 0.05%. The level of evidence assigned to these studies is “fair”.

### 3.7 Art Therapy

Art therapy is a term which encompasses a large set of terms for dance, movement, music^1^, art, theater, yoga and poetry.

Regarding the impact of art therapy (AT) on the well-being of children in oncology, only two quantitative studies are available (Favara-Scaco et al., 2001; Madden et al., 2010). Other studies regarding AT for the pediatric oncology population are more qualitative and indicate having observed subjective improvements in patients (Walker, 1989; Gunter, 2000; Rollins, 2005). On the other hand, examples of quantitative studies of the impact of art therapy are much more numerous in the adult population (Geue et al., 2010) or the pediatric population not suffering from cancer (Beebe et al., 2010). These studies report an improved quality of life and reduced anxiety and depression. However, these studies are often small (n<50) and rarely randomized, which limits the scope of their evidence (Geue et al., 2010).

In the study by Madden et al. (2010), the sampling method is mixed: a part is from a randomization (n=16) and the other not (n=32). The intervention consists of six one-hour sessions of AT (2 in dance/movement, 2 in music and 2 in graphic arts) followed over six infusion sessions in children from the ages of 2 to 21 years. The control group receives assistance from a volunteer to speak, read or watch TV. In the randomized group, it appears that the effect of art therapy is significantly positive with respect to the child’s pain (p=0.03) and taste for food (p=0.0061) during chemotherapy, compared to a group without art therapy. In the non-randomized group, the measurement instruments used are different and show a significant and positive impact of art therapy in regard to facial expression (measured by “The Faces Scale’) (p<0.01), the excitement of the patients (p<0.05), happiness (p<0.02) and nervousness (p<0.02).

In the experimental study with historical controls by Favara-Scaco et al. (2001), 32 children between the ages of 2 and 14 were treated with bone marrow aspirations or lumbar puncture. AT methods before, during and after the punctures were as follows: clinical dialogue to calm children and help them cope with painful procedures; visual imagination to activate alternative thought processes; medical game to clarify the illness, eliminate doubts, and offer greater control over a threatening reality; structured drawing to contain anxiety by offering a structured framework controllable by children; free drawing to allow children to act out their confusion and fears, and theater to help children come to terms with their changing bodies. The patients in the historical control group demonstrated resistance behavior and anxiety during and after painful procedures (82%). In contrast, children who received one session of AT from the first hospitalization exhibited collaborative behavior (72%). Parents also expressed being able to better manage painful procedures where art therapy was provided. No statistical tests, however, are provided.

In summary, there are many studies reporting improved quality of life and reduced anxiety and depression through art therapy in pediatric and adult patients in general. However these studies use small sample sizes (n<50) and are rarely randomized, leading to a level of evidence that is “fair”. Among the studies listed, only two studies evaluated the impact of art therapy on the well-being of children with cancer. In pediatric oncology, art therapy would have a significantly positive effect on pain, facial expression, excitement, happiness, nervousness and anxiety.

### 3.8 Music Therapy

Music therapy is not the simple act of listening to music. This is a more participatory activity in which the patient is engaged in a creative process. The definition that can be given is that of a ’live’ music carried out with a music therapist and which may take on various forms: improvisation, songwriting, remake of a song, etc. It aims to improve the physical well-being and mental health of the individual facing a serious illness and its treatment.

The specific mechanisms behind the therapeutic effects of music are difficult to determine. Some theories describe the action of music on the nervous system as a pituitary stimulation with release of endorphins. Another explanation suggests an increase of catecholamines leading to a reduction in heart rate and blood pressure (Henry, 1995; Whipple and Glynn, 1992). Moreover, it would appear that the cardiovascular and respiratory effects of the music would also be linked to the musical rhythm used. This is indeed what is suggested by the study by Bernardi et al. (2006) by comparing different styles of music.

Many studies on the beneficial effects of music therapy are available. These relate to both children and adults. However, regarding pediatric oncology specifically, the number of these studies is considerably reduced. We have thus identified five studies, including three which are randomized: Colwell et al. (2005), Robb et al. (2008) and Bufalini (2009).

The first of these randomized studies, by Colwell et al. (2005), compares art therapy (drawing) with music therapy (playing an instrument) during a hospital stay. The results of 24 pediatric patients, aged 7-18, showed no significant positive difference between the two therapies on the scale measuring psychological health called “Piers-Harris Children’s Self Concept.” By comparing the results before and after therapy in each group, there is an improvement in both groups, but only art therapy is significantly positive (p<0.05). In turn, the randomized study by Robb et al. (2008) with 83 patients indicated a significant difference in favor of music therapy, compared to merely listening to music (control group 1) or listening to a recorded story (control group 2), as regards to facial expression (p<0.001) and engagement (p<0.001) in children aged 4 to 7. Regarding interaction with their environment, a better result is obtained with music therapy or simply listening to music over listening to a recorded story (p<0.05). Finally, the third randomized study that we identified, by Bufalini (2009), conducted among 39 patients undergoing a painful medical procedure (lumbar puncture, bone marrow aspiration, bone marrow biopsy and arterial catheter), indicated a decrease in anxiety and greater collaboration from the group of children receiving conscious sedation and music therapy, in comparison with the control group receiving complete sedation (p<0.05).

The two non-randomized studies that we have identified also support the idea of a beneficial effect of music therapy for patients in pediatric oncology. In the pre-and post-therapy study by Barrera et al. (2002) (n=65), interactive music therapy has reduced anxiety and pain and increased patient engagement in game activities during their hospitalization (p<0.01). The results provided by the children are consistent with the impression from parents and care givers (Barrera et al., 2002). Case studies by O´Callaghan et al. (2007), conducted in a radiation oncology waiting room, also report lower stress and an increase of communication in children after an experience of active music therapy.

Another randomized study, by Caprilli et al. (2007), although not conducted in children with cancer, may also be of interest to the extent that it addresses the issue of pain during a venipuncture. The results of this study indicate that the use of music therapy significantly reduces pain and distress caused by venipuncture in comparison with a control group without music therapy (p<0.05 and p<0.001).

Apart from these few studies specific to pediatric oncology, we must also mention the existence of several systematic reviews and meta-analyses evaluating the effects of music therapy on pediatric patients. In particular, the systematic review by Mrazova and Celec (2010) indicates a large heterogeneity in randomized studies conducted both in regard to the type of intervention (active or passive music therapy, individual or group, etc.) and the category of pediatric patients involved (treated for depression, autism, etc.) or the control group used. The results are generally positive: 23 of 28 studies reported a beneficial effect on the well-being of patients. Mrazova and Celec (2010) mention that music therapy can affect well-being in many ways: anxiety, stress and pain perception can be reduced during invasive medical procedures, symptoms of psychiatric disorders such as schizophrenia, depression, autism and others can be alleviated, cognitive skills of communication can be improved, and improved sleep or feeding can be achieved in premature infants. It is also worth mentioning the meta-analysis by Standley and Whipple (2003) conducted on 29 observational studies conducted during invasive medical and non-invasive procedures and which concluded that music therapy reduces pain, anxiety and distress in children. Similarly, the meta-analysis by Klassen et al. (2008) indicates a small to medium effect of music therapy in the context of 19 randomized studies conducted during medical procedures.

In summary, interactive music therapy in which the child is actively involved in the creation of music has a beneficial effect on the well-being of children with cancer, and in many ways: reduction of anxiety, distress, stress and pain, general behavior of the child. The number of studies reported in pediatric oncology, however, is reduced, indicating an overall level of evidence as “fair”.

## 4. Discussion

The review of literature carried out here, regarding the effects of CAM on the well-being of pediatric oncology patients, particularly highlights the plurality of CAM and the diversity of methods used to test their effectiveness. In fact, the control groups, as well as the strategy developed within each CAM, are almost always different from one study to another. Furthermore, in a number of studies, not only one CAM is involved, but rather various CAM, which prevents determining the true source of the effect observed on the well being of the children, especially when it comes to studies of distraction and imagery. Moreover, the practices of CAM are frequently customized to the needs of the child, so it is common for two patients not to receive the same treatment although they belong to the same treatment group. This feature is however inherent to the practices of CAM as mentioned by Landier and Tse [2010, p. 578]: “Although mind–body interventions for management of procedure-related symptoms in pediatric oncology may be effective, particularly when used in combination with pharmacological agents, individual differences in age, temperament, and prior procedure-related experiences make it imperative for nurses to conduct thorough patient assessments to determine the most efficacious interventions for individual patients.” However, a thorough assessment of each patient could sometimes be difficult since it requires not only knowing the child, but also knowing the different CAMs and their optimal use.

Apart from these first points, many other issues have also emerged from reading the studies reported, such as the lack of description of the method of randomization, the reasons for attrition, the lack of clarity in the definition of the nature of the intervention (who does what and when) or the lack of description of the characteristics of the patient. It is also very common to note the small sample size, which limits the scope of the results and increases the risk of sampling bias. All these weaknesses naturally lead one to take a prudent look at the results recorded. However, very few studies suggest a lack of positive effects and none indicates an opposite effect to that intended. This last point may be due to the observation of a real effect, but also that of a publication bias. Consequently, the recommendations made in the rest of this article cannot be regarded as strong as they are mainly based on trends of small sized studies with a significant number of biases for most of them. However, the same trends are also observed in pediatric populations other than in oncology and in adult populations, it is thus likely that the positive effects of CAM are real in pediatric oncology, at least for some of them.

**Table 1:** Summary of studies on the pediatric oncology population

CAM	Number of studies	Level of Evidence	Overall Results	Resources
Distraction	Two meta-analyses	Good to fair	↓ pain	Closed room
	7 RCT		↓ anxiety	Therapist
	9 NRCT		↓ distress	
			↑ control	
Imagery	2 RCT	Fair	↓ pain	Closed room
	4 NRCT		↓ anxiety	Therapist
			↓ distress	
Hypnosis	1 meta-analysis	Good to fair	↓ pain	Closed room
	14 RCT		↓ anxiety	Hypnotherapist
	1 NRCT		↓ distress	
			↓ fear	
			↓ nausea	
			↓ vomiting	
Massage Therapy	3 RCT	Fair	↓ anxiety	Closed room
			↓ heart rate	Massage therapist
			↓ discomfort	Massage table or chair
			↓ pain (muscle)	
			↑ breathing	
Aromatherapy	1 RCT	Fair	↓ pain	Oil diffuser
			↓ nausea	
Chiropractic	0	Low	↓ pain *	Closed room
			↑ quality of Life *	Chiropractor
				Dedicated table
Acupuncture	2 RCT	Fair	↓ vomiting	Closed room
	1 NRCT		↓ antiemetics	Acupuncturist
			↑ relaxation	
Art Therapy	1 RCT	Fair	↓ pain	Closed room
	1 NRCT		↓ nervousness	Art therapist
			↑ well-being	Activity cart
			↑ taste (food)	
			↑ cooperation	
Music Therapy	3 RCT	Fair	↓ anxiety	Closed room
	2 NRCT		↓ pain	Music therapist
			↑ game activity	Activity cart
			↑ well-being	
			↑ communication	
			↑ engagement	

* : By extrapolation from other studies with children.

Studies reported in this systematic review indicate very few adverse events, indicating that CAM is relatively safe. However, a list of contraindication should be established for all management strategies and ideally on an individual basis to avoid any adverse effect. In this setting, communication between patients and health professionals is essential. This communication could be done in line with the rationale for the use of CAM in pediatric oncology patients. The reasons are generally as follows (Paisley et al., 2011; Bishop et al., 2010): 1) to improve overall health and well-being (50-78%); 2) to cure, prevent or slow the progression of cancer (11-59%); 3) to reduce the symptoms related to cancer (25-33%); 4) to reduce the side effects of traditional treatments (11-30%). Moreover, in the study by Paisley et al. (2011), 80% of users of CAM consider them as effective or very effective.

Finally, we should mention that a limit of our systematic review is that for some CAM, very few studies were available, which led us to indirectly consider the result of other studies with children without cancer. Sometimes, we also addressed studies with adults with cancer, but it was only to offer a more comprehensive view of the effect of a specific CAM; indeed, these studies were not considered to establish the global level of evidence. We should also mention that it was in some cases hard to compare different design of studies, as a consequence we gave more importance on RCT. Finally, we did not consider the effect of CAM as regard to cancer stage, actual diagnosis, age and gender, which could be a limit of our systematic review.

## 5. Recommendations and Conclusion

Given the favorable results obtained with hypnosis compared to distraction, and the number of available studies, this CAM is the one most likely to be recommended for inclusion in the offer of health service of establishments with a pediatric oncology department. However, it will be necessary to evaluate the sensitivity of children to hypnosis before any intervention; the less sensitive children will then turn to other CAM already offered at their hospital. These other CAM could be art therapy and music therapy, which can be regarded as more advanced forms of distraction and imagery. Indeed, both CAMs showed potential to improve the well-being of patients.

Given the results obtained with massage therapy (the only large randomized study has not led to any difference in outcome), we cannot make a clear recommendation. The same is true for aromatherapy. Based on available data, chiropractic and osteopathy cannot be recommended. Similarly, with regard to acupuncture, although early results are encouraging, there are too many uncertainties on the acceptance of such technology by children and their families, as well as the optimal procedure suitable for children. We therefore cannot recommend it.

Ultimately, it seems that CAMs are potential and desirable avenues for the improvement of the health and well-being of children being treated for cancer. However, in order to strengthen their presence in health facilities, these must be better supported by randomized quality studies and the intervention protocol followed be more explicit in order to determine the best practices. Similarly, studies comparing more than two CAMs should also be conducted.

## Footnotes

The studies on music therapy only are reported in the next section.
